# Validation of astrocytic reference genes for qRT-PCR in CO treatment studies

**DOI:** 10.1186/2193-1801-4-S1-P32

**Published:** 2015-06-12

**Authors:** Sara Oliveira, Helena Vieira, Carlos Duarte

**Affiliations:** Center for Neuroscience and Cell Biology, University of Coimbra, Portugal; Chronic Diseases Reasearch Center FCM, Portugal

**Keywords:** Carbon monoxide, astrocytes, reference genees

Quantitative real-time reverse transcription-polymerase (qRT-PCR) is a widely used technique to characterize changes in gene expression in complex cellular and tissue processes, such as the cytoprotection or inflammation. The selection of an adequate internal reference gene for accurate and consistent analysis of gene expression is of major importance. Carbon monoxide (CO) affects several metabolic pathways and de novo protein synthesis is crucial in the cellular responses to the gasotransmitter. Herein a selection of commonly used reference genes was analyzed to identify the most suitable internal control genes to evaluate the effect of the CO on gene expression in cultured cortical astrocytes. The cells were exposed to CO by incubation with CORM-A1 (CO releasing molecule A1) and four different algorithms (geNorm, NormFinder, Delta Ct and BestKeeper) were applied to better evaluate the stability of eight putative reference genes. Our results indicate that Gapdh (glyceraldehyde-3-phosphate dehydrogenase) and Ppia (peptidylpropyl isomerase A) is the most suitable gene pair for normalization of qRT-PCR results under the experimental conditions used. Pgk1 (phosphoglycerate kinase 1), Hprt1 (hypoxanthine guanine phosphoribosyl transferase I), Sdha (Succinate Dehydrogenase Complex, Subunit A), Tbp (TATA box binding protein), Actg1 (actin gamma 1) and Rn18s (18S rRNA) genes presented less stable expression profiles in cultured cortical astrocytes exposed to CORM-1 for up to 60 min. Analysis of the effects of CO on the expression of Bdnf and bcl-2 gave different results depending on the reference genes used. A significant increase in the expression of both genes was found when the results were normalized with Gapdh and Ppia, in contrast with the results obtained when the other genes were used as reference. This study highlights the need for proper and accurate usage of reference genes in quantification of qRT-PCR results in studies on the effect of CO in gene expression.

